# Intestinal anastomotic healing models during experimental colitis

**DOI:** 10.1007/s00384-021-04014-5

**Published:** 2021-08-28

**Authors:** J. R. E. Miltschitzky, Z. Clees, M.-C. Weber, V. Vieregge, R. L. Walter, H. Friess, S. Reischl, P.-A. Neumann

**Affiliations:** 1 School of Medicine, Klinikum Rechts Der Isar, Department of Surgery, Technical University of Munich, Munich, Germany; 2 School of Medicine, Klinikum Rechts Der Isar, Department of Diagnostic and Interventional Radiology, Technical University of Munich, Munich, Germany

**Keywords:** Anastomotic leakage, Insufficient healing, Dextran sodium sulfate

## Abstract

**Background:**

Anastomotic leakage represents a major complication following resections in colorectal surgery. Among others, intestinal inflammation such as in inflammatory bowel disease is a significant risk factor for disturbed anastomotic healing. Despite technical advancements and several decades of focused research, the underlying mechanisms remain incompletely understood. Animal experiments will remain the backbone of this research in the near future. Here, instructions on a standardized and reproducible murine model of preoperative colitis and colorectal anastomosis formation are provided to amplify research on anastomotic healing during inflammatory disease.

**Methods:**

We demonstrate the combination of experimental colitis and colorectal anastomosis formation in a mouse model. The model allows for monitoring of anastomotic healing during inflammatory disease through functional outcomes, clinical scores, and endoscopy and histopathological examination, as well as molecular analysis.

**Discussion:**

Postoperative weight loss is used as a parameter to monitor general recovery. Functional stability can be measured by recording bursting pressure and location. Anastomotic healing can be evaluated macroscopically from the luminal side by endoscopic scoring and from the extraluminal side by assessing adhesion and abscess formation or presence of dehiscence. Histologic examination allows for detailed evaluation of the healing process.

**Conclusion:**

The murine model presented in this paper combines adjustable levels of experimental colitis with a standardized method for colorectal anastomosis formation. Extensive options for sample analysis and evaluation of clinical outcomes allow for detailed research of the mechanisms behind defective anastomotic healing.

**Supplementary information:**

The online version contains supplementary material available at 10.1007/s00384-021-04014-5.

## Introduction

Delayed or insufficient healing at the site of a colorectal anastomosis can lead to anastomotic leakage (AL) with the respective morbidity. With worldwide incidence rates of 1–24% [1–6], it is a frequent and serious complication leading to intraabdominal septic conditions and even death. A wide variety of animals (ranging from mice and rats to dogs and pigs) are used, and a large number of risk-factors and therapeutic approaches for AL have been examined [7].

Although the discussion around the most appropriate surgical technique is still ongoing [8–11], it is well accepted that precise adaption of the tissue, especially the serosa, is an indispensable requirement for anastomotic healing. However, even in case of a technically flawless anastomosis formation, several risk factors remain a threat to the healing process by influencing the immune response or tissue perfusion. Even in case of a technically flawless anastomosis, several risk factors are threatening the healing process by influence on the immune response or perfusion. The underlying (patho-)physiology of anastomotic healing consists of an inflammatory, a proliferative, and a regenerative phase [12, 13]. Transition between these phases is smooth, and it is important to see them as part of one intricate, orchestrated continuum. Overwhelming inflammation such as in inflammatory bowel disease (IBD) hampers the anastomotic tissue from proceeding to the later phases of wound healing [14]. The dilemma in treatment of patients with inflammatory bowel diseases (IBD) is that they are prone to operations while at the same time requiring immunosuppressive medication. This corroborates the call for development of further treatment strategies in this high-risk cohort.

A vast amount of approaches has been evaluated. However, despite several decades of focused research, no clinically approved therapy to markedly reduce the incidence of AL has been translated into clinical routine yet [15].

This only highlights the need for dedicated research in order to fill the gaps in our knowledge of how the complex gastrointestinal healing processes work – and how AL especially in chronic inflammatory conditions – can be treated or even prevented altogether. To this date, pre-clinical research on anastomotic healing and colitis is often poor in quantity and quality of reporting [7]. Hence, the aim of this project is to demonstrate a standardized, reproducible, and easy-to-learn murine model combining experimental colitis with colorectal anastomosis formation and to present several scores to analyze anastomotic healing on a functional, macro-, and microscopic and molecular level. This should contribute to more standardized and therefore better quality research in the future.

## Methods

In the following, a brief and concise description of the experimental setup will be outlined. For more detailed and complete information, we refer to the supplemental material.

### Compliance with regulations for animal experiments statement

All procedures that are performed on animals should be conducted under specific pathogen-free (SPF) conditions as defined by FELASA and must be approved after legal and ethical review by the local animal welfare committee of the performing institution.

### Animal experiments

It is recommended to use adult female mice at an age of 10–12 weeks and with an average weight of 20 g. Although the use of only one sex in an animal experiment is usually discouraged, females, unlike the more aggressive males, can be co-housed. This facilitates equalization of commensal gut microbiota and thus helps to standardize the experimental conditions. It is important to closely monitor the mice clinically for weight loss and to assess pain and distress daily. Before the start of the experiments, mice which are not bred in-house need a standardized acclimatization phase to adapt to the local microbiota of animal housing and eliminate confounding factors like stress associated with shipping. At least 7 days are recommended for this phase, but duration can be adapted to local conditions. If possible, use co-housing to improve exchange of commensal bacteria and include the same acclimatization phase in the protocol for control groups to ensure comparable results.

### Colitis induction

In the following, two different methods for induction of chemically induced colitis in mice will be described.

**Fig. 1:**
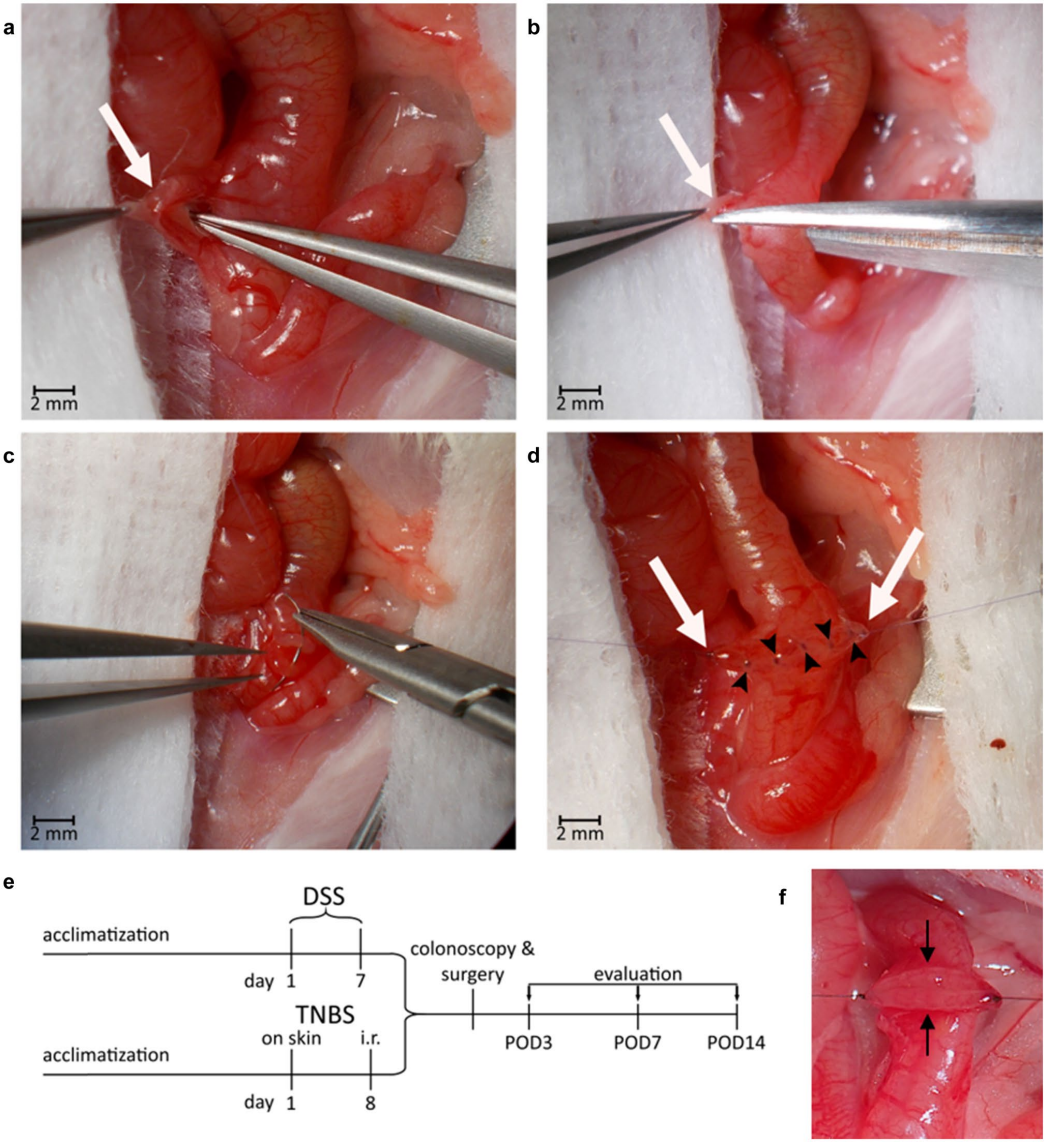
Illustration of the surgical procedure. **a–d** Selected steps of surgery on anesthetized mouse in supine position after median laparotomy. Photographed through operating microscope. **a** Blunt separation of the mesocolon (white arrow) with mesenteric vessels from the intestine after the large and small intestine have been mobilized. **b** Vessel-sparing dissection of the colon. **c** Placement of first suture to adapt the two colonic ends. In total one mesenteric and one antimesenteric holding suture are tied. **d** Completed anastomosis before cropping the holding sutures (white arrows). Between them, the dorsal row of five single stitches (black arrowheads) can be seen. Final anastomosis made up of a total of 12 full thickness single stitches (2 holding sutures, 5 anterior, and 5 posterior stitches). **e** Overview of the experimental timeline. The experiment starts with an acclimatization phase followed by induction of colitis. DSS colitis is induced by continuous exposure to DSS in ad libitum drinking water. TNBS colitis is induced by rectal administration of TNBS 7 days after cutaneous presensibilization. In both colitis models, this phase is followed by colonoscopy to assess colitis and integrity of the colon, then by surgery, both under general anesthesia. Mice are allowed to recover while monitored, scored, and provided with pain medication until evaluation. Evaluation can take place on postoperative day 3 (POD3), POD7, or POD14 depending on the focus of research being early or late healing phases. **f** Everted mucosal layer after transection of the colon (black arrows)

For dextran sodium sulfate (DSS) colitis (Fig. 1e), we refer to the established protocols [16]. In brief, DSS is dissolved in standard drinking water to a set percentage depending on the acquired intensity of inflammation (see below). This water is then offered ad libitum for several days to induce colitis. In terms of the timeframe and dose for induction, it is best to aim for mild colitis (target disease activity index (DAI) [16] of 1–2), in order to ensure an inflamed, but not completely ulcerated mucosa. This prevents excessive weight loss, high disease activity, and high strain on the animals. We recommend to start with colitis induction 7 days preoperatively. After surgery, mice are offered normal water. It is important to note that the dose of DSS varies widely depending on environmental factors.

To induce 2,4,6-trinitro benzene sulfonic acid (TNBS) colitis (Fig. 1e), we adapted the protocol by Wirtz et al. [16]. Pre-sensitization is performed 7 days prior to surgery under inhalational anesthetic (e.g., isoflurane). A 1 × 1 cm patch of skin is shaved on the back of the mouse, and the freshly mixed pre-sensitization solution is applied. We adapted the protocol using only 30 µl of pre-sensitization solution to allow for complete absorption. It is important to make sure the same amount of fluid is used on every individual animal.

After the 7 days of pre-sensitization, TNBS colitis is induced by rectal application of the induction solution. To do so, gently advance a 3.5 F catheter into the colon until a soft resistance (the colonic flexure) can be felt, making sure not to damage the colon in the process. Slowly inject 50 µl of the solution and hold the mouse head down for 1 min. The effect of TNBS colitis depends on the mouse strain; the microbiotic and genetic factors and therefore disease activity can vary. Adapt the dosage of TNBS for weight loss of less than 5% on the day after induction. It is recommended to abort the experiment for an individual animal if this limit is exceeded.

In both models, colonoscopy can be used to assess presence of inflammation and verify integrity of the colon after inducing colitis before proceeding to surgery.

### Anesthesia and analgesia

It is recommended to use inhalational (e.g., isoflurane) over injectable anesthetics (e.g., medetomidine, midazolam, and fentanyl) to prevent loss of its effects during the procedure. Although initial induction by intraperitoneal anesthesia is imaginable, it is not safely possible to boost anesthesia intraoperatively or antagonize postoperatively.

Since isoflurane does not provide an analgesic effect sufficient for surgical tolerance, additional analgesics must be administered. In total, 1 mg/kg of metacam s.c. and 0.1 mg/kg buprenorphine s.c. about 20 min prior to isoflurane application should be administered. Administer an additional dose of 0.1 mg/kg buprenorphine s.c. about 6 h after surgery and 1 mg/kg of metacam s.c. each on the first and second postoperative day. Note, however, that NSAIDs like metacam can interfere with inflammatory components of the model and opioids like buprenorphine decrease bowel motility.

Induce general anesthesia for colonoscopy, surgery, and euthanasia. Apply eye ointment to protect the cornea. Avoid hypothermia by using an adjustable heating pad.

### Colonoscopy

**Fig. 2:**
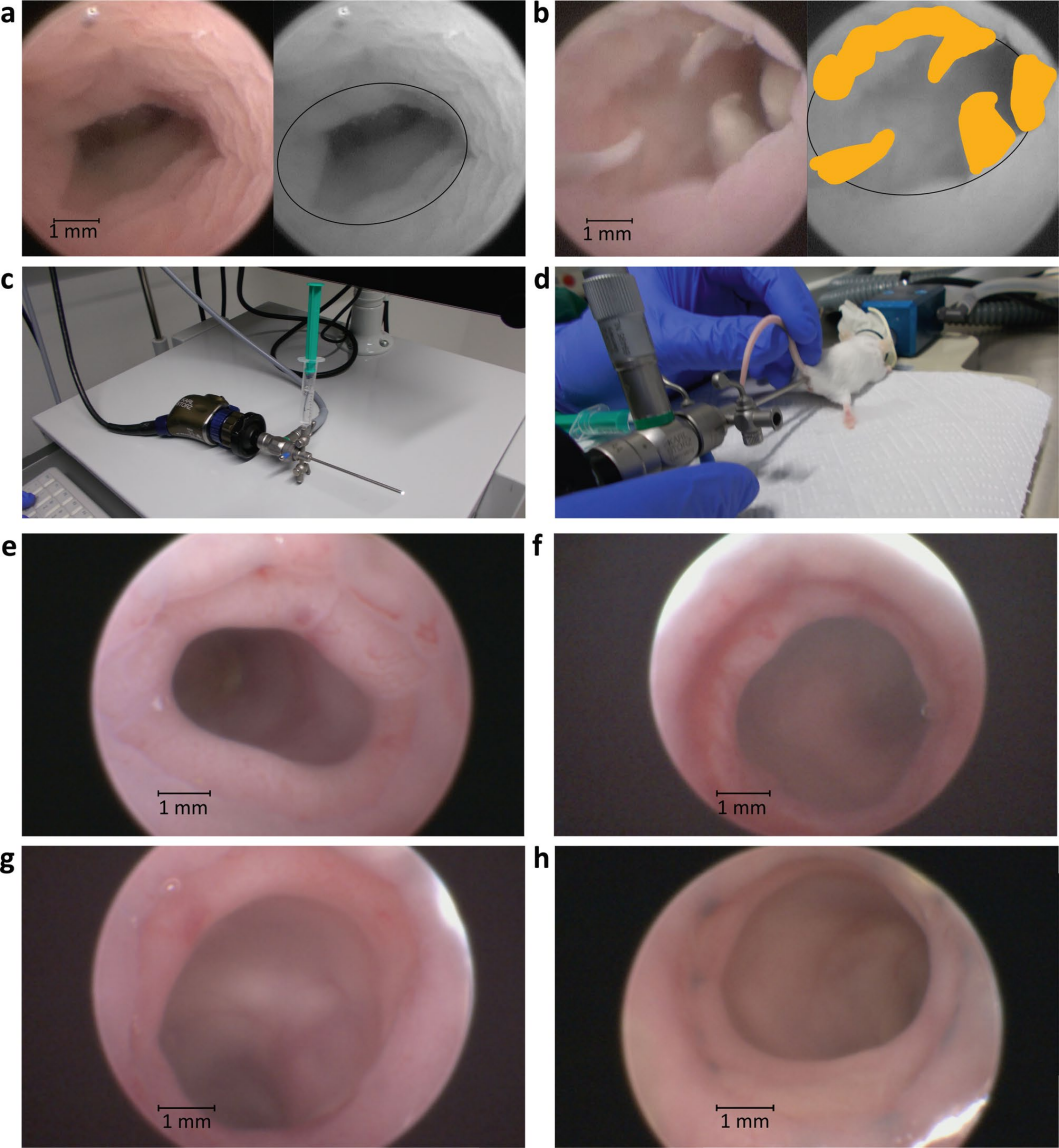
Endoscopic scoring and evaluation of the anastomosis. **a, b** Colonoscopies after surgery on POD7 using sodium chlorine solution as examples for endoscopic scoring. Original image on the left, monochrome image with ellipsoid as a marker for circumference at the site of anastomosis and fibrin marked in yellow on the right. **a** Anastomosis with no evidence of any dehiscence, yielding an endoscopic score of 0. **b** Endoscopic score of 3 with fibrin protruding into the lumen at more than one quarter of the circumference of the colon. **c** Colonoscope with attached light cable and 10-ml syringe for water-assisted colonoscopy. **d** Colonoscopy on anesthetized mouse in prone position. **e–h** Colonoscopies using air as a medium. **e** Well healed anastomosis on POD3 compared to **f** anastomosis at POD3 with intraluminal bleeding around most of the circumference. **g** Well healed anastomosis on POD7 compared to **h** anastomosis on POD7 with a large fibrin patch at 6 o’clock

Place the anesthetized mouse prone onto a heated pad; limbs may be fixated with tape. Insert a murine endoscope (e.g., Image 1 HD SCB 22,201,020 hub, Xenon 175 20,132,120 light source, H3-Z 22,220,055 camera head, HOPKINS® 64,301 AA straightforward telescope 0° and 61,029 C trocar, all by KARL STORZ & Co. KG., Fig. 2c) under careful insufflation to minimize trauma to the anus, while keeping the tail in one hand (Fig. 2d). Lubrication might be used to facilitate this process.

Endoscopy can be performed using either air or 0.9% sodium chlorine solution for insufflation. Sodium chlorine solution allows for gentler insufflation and a very even distribution of pressure along the colon, whereas the air as a medium allows for clearer images (see Fig. 2a, b, e–h). To evaluate the anastomosis, advance the endoscope up to the colic flexure, and then start recording a video while pulling back steadily until exiting the colon for standardized documentation. Gently rotate the endoscope at the site of anastomosis to get a good visual of the entire perimeter of the wound.

### Colorectal anastomosis formation

In the following, vessel sparing anastomosis formation without bowel resection is described to minimize the potential influence of ischemia on the healing process. In brief, place mouse in supine position, access the colorectum via median laparotomy and blunt preparation technique, and transect the colon between two vasa recta while taking great care not to injure the mesenterial artery (Fig. 1a, b). This vessel along with its branches must be spared to preserve blood supply to the colon and avoid confounding the experiment with ischemic components of anastomotic leakage. Reconnect the colonic stumps in an end-to-end fashion and adapt the suturing technique to the requirements of the experiment. More stitches and running sutures provide tighter approximation of the anastomotic region. To get a higher rate of AL, the total number of stitches can be reduced to eight or even less, while twelve single sutures usually guarantee a very low leakage rate (see Fig. 1c, d for an example with twelve single stitches). Monofilamentous, resorbable polyglactin or polydioxanone sutures are used in humans and can be recommended for this surgical model, also. In proportion with the suture diameter used in human patients, 11–0 would be the analogue suture size in the murine intestine, but this suture is expensive and difficult to handle due to its small size. A 9–0 suture represents a good trade-off between size, price, and handling. Note that this model per se constitutes one of defect healing due to the fact that the mucosa is everted because it is technically almost impossible to attach the serosa layers (Fig. 1f).

### Postoperative management

Score mice daily after surgery. We recommend a scoring protocol including the criteria listed in Table 1. We have defined an algorithm for therapeutic measures as well as clear abort criteria based on this score: a total score of 3 requires substitution of i.p. fluid and analgesia; we have aborted the experiment at a total of 8 score points or any single score of 4 or above. Pay special attention to proper healing of the skin suture; revise the suture under general anesthesia if necessary. To improve postoperative weight recovery, oatmeal soaked with glucose solution can be added to the diet. During the early postoperative phase, therapeutic measures, like analgesic treatment by s.c. injection of 1 mg/kg metacam, water soaked food, hydrogel, and substitution of fluid by s.c. injection of 0.9% sodium chloride solution, may be applied.

**Table 1:** Score sheet

**Criteria**	**Score points**
Weight	0 = no reduction
	1 = reduction of 0–5%
	2 = reduction of 6–10%
	3 = reduction of 11–19%
	4 = reduction of > 19%
Fur	0 = normal, shiny, smooth
	1 = piloerection
Behavior	0 = normal
	1 = subdued, no exploration, reduced interaction
	4 = apathy, isolation, stereotypic behavior
Posture	0 = normal
	2 = intermittent cowering or shivering
	4 = permanent cowering or shivering
Pain	0 = no indication
	2 = defensive behavior on palpation of abdomen
Impaired wound healing	0 = no indication
	2 = red or oozing wound
	4 = dehiscent suture or ruptured abdomen
Dehydration	0 = skin folds straighten within 2 s
	1 = persistent skin folds
Mucous membranes (ears, skin, extremities)	0 = rosy
	1 = pale
Stool	0 = formed
	1 = diarrhea

### Evaluation of anastomotic healing

Depending on the experimental setup and on the scope of the experiment, different days might be considered for evaluation for the healing process. It is useful to evaluate mice on postoperative day 3 (POD3) for early healing and POD7 and POD14 for late healing. Perform in vivo colonoscopy in the anesthetized animal to score endoscopic healing (Fig. 2a, b, e–h). Sacrifice the mouse, perform longitudinal laparotomy, and grade adhesions and abscess formation by mobilizing the anastomosis and bluntly removing adhesions wherever possible without compromising the integrity of the anastomosis. Extract the colon containing the anastomosis from rectum to the right colic flexure, and gently clear it of feces with a cotton swab.

#### Bursting pressure measurement

To measure bursting pressure, mount the extracted large intestine onto a petri dish, and insert a plastic cannula into the aboral lumen and an ICP pressure probe (e.g., Omnibar E5F probe and MPR Datalogger, Raumedic AG) into the oral lumen, and fixate with polyfilamentous 4–0 ligatures. After calibrating the pressure in the colon to 0 mmHg, fill it with isotonic saline solution using a syringe attached to the cannula until rapid pressure drop while constantly measuring the intraluminal pressure. Record the maximum of the pressure spike as bursting pressure. Additionally, document the bursting location, differentiating between bursting location at anastomosis or not at anastomosis. This technique provides an easily measurable parameter of mechanical stability independent of microscopic healing.

#### Collection and preparation of biological samples

**Fig. 3:**
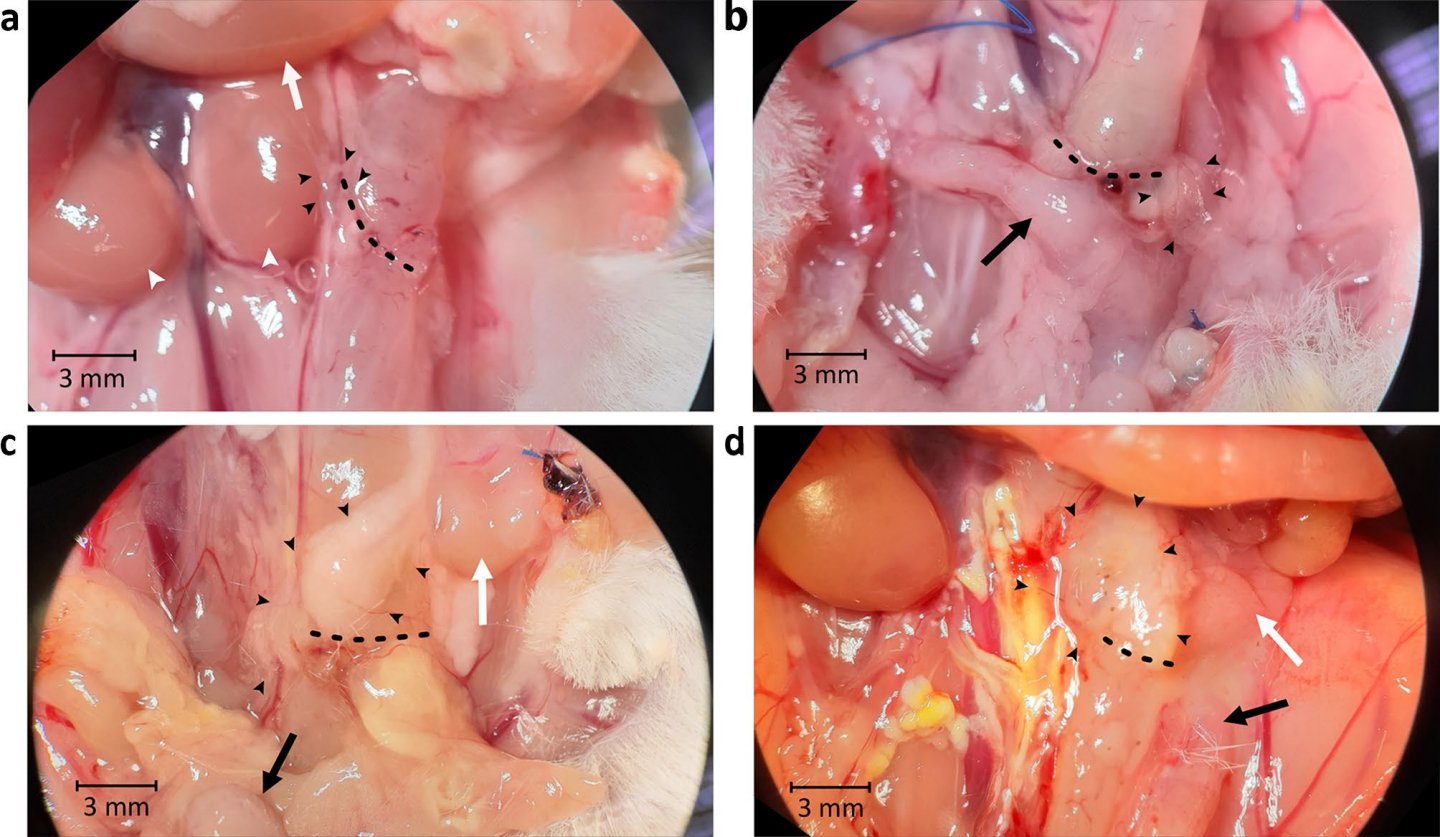
Macroscopic scoring of the anastomosis. **a**–**d** Evaluation of anastomosis in situ. Dotted line: anastomosis; black arrowheads: pancreas and fat adhesions; black arrow: uterus; white arrow: small intestine; white arrowheads: kidneys. **a** Score of 2a. A bit of omentum (1 point), but neither the small intestine, uterus (not in the picture), nor any other organ such as a kidney adhere (0 point each). This adhesion could be removed completely (1 point). There is no visible abscess or peritonitis in the abdominal cavity. **b** Score of 3a. Uterus and some omentum are stuck to the anastomosis (1 point each), but neither the small intestine (not in the picture) nor any other organ (0 points). All adhesions could be removed (1 point). There is no visible abscess or peritonitis in the abdominal cavity. **c** Score of 5a. Omentum and small intestine stick to the anastomosis (1 point each), but no other organ such as the uterus (0 point). Adhesions could not be removed without compromising anastomosis integrity (3 points). There is no visible abscess or peritonitis in the abdominal cavity. **d** Score of 6a. Omentum, small intestine, and uterus are stuck to the anastomosis (1 point each), but no other organ (0 point) and all adhesions could not be removed bluntly (3 points). There is no visible abscess or peritonitis in the abdominal cavity

There are multiple options to collect and analyze tissue samples. Feces can be retrieved for microbiological analysis. To collect tissue samples, the colon should be placed on a petri dish and dissected lengthwise along the mesocolon. Then, the flattened colon can be dissected lengthwise again with a 22-blade scalpel. We recommend using one-half for biochemical analysis of the tissue, the other half for histology. Use a biopsy punch to retrieve samples from different regions of the colon. For histology, flatten the other half of the colon on the petri dish. To prevent it from coiling up, pipette tissue fixative directly onto the tissue. After a few minutes, place colon into a tube; store tube horizontally until dehydration and embedding. Refer to Fig. 3 for an illustration on sample preparation.

### Applicable scores

#### Endoscopic score

To quantify anastomotic dehiscence and evaluate healing through colonoscopy, we have defined a score ranging from 0 to 4.

See Table 2 for the score and Fig. 2a, b for demonstration of the application of this score. We propose to use this score to assess macroscopic mucosal healing and gross morphology of the anastomosis in vivo. In addition, the degree of dehiscence can also be measured and quantified [17], and if applicable, fluorescent endoscopy can be used for evaluation of different markers of the healing process.

**Table 2:** Endoscopic healing score

**Score points**	**Criteria**
0	No dehiscence
1	Suture thread protruding into lumen
2	Slight dehiscence, necrotic tissue, or fibrin on less than a quarter of circumference
3	Advanced dehiscence, necrotic tissue, or fibrin on more than a quarter of circumference
4	Full dehiscence, visible hole into the peritoneal cavity

#### Histological score

**Fig. 4:**
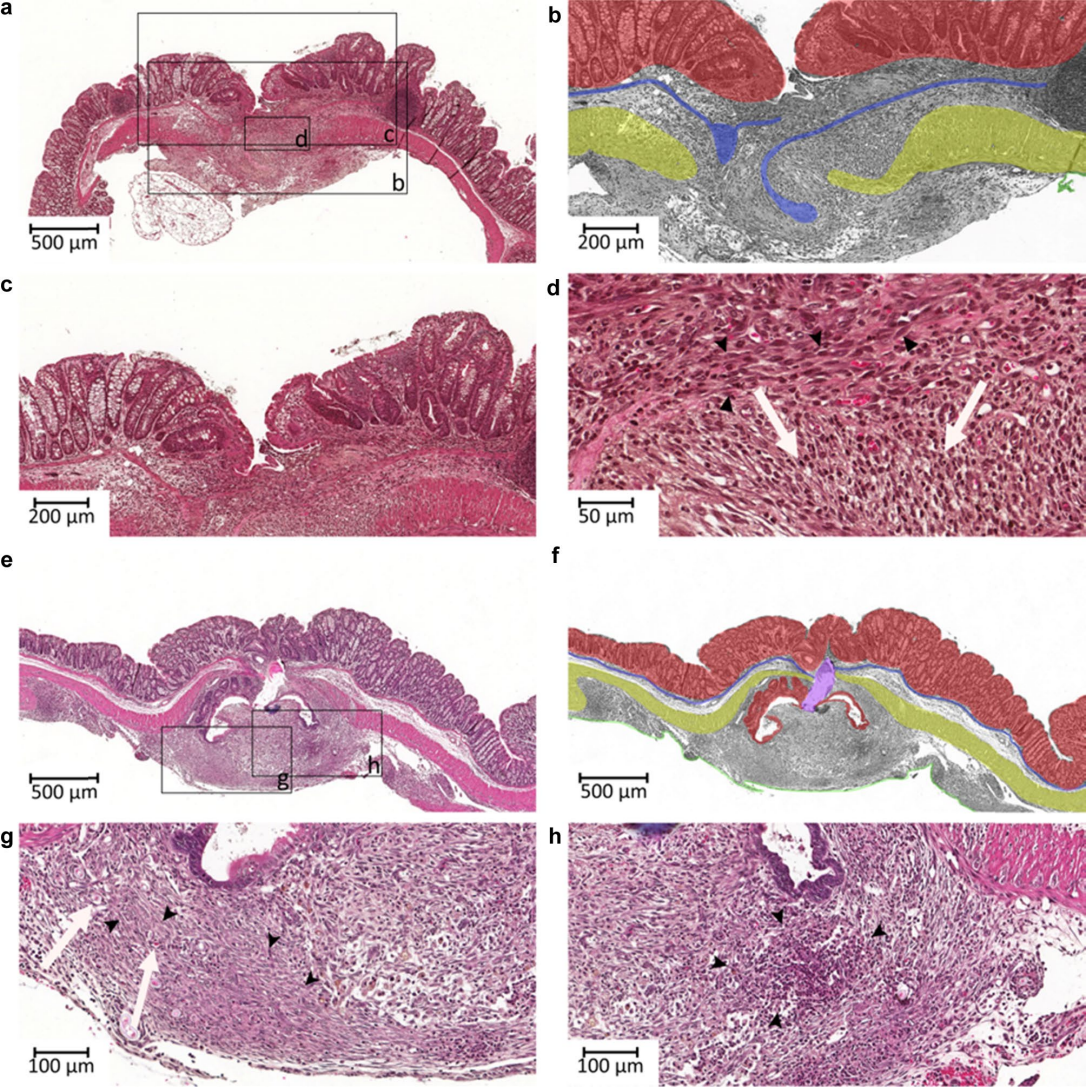
Histopathological scoring. Exemplary histological sections of anastomoses with histological score explained in the following, compared to Table 2. **a** Example of a poorly healed anastomosis: overall healing has been graded bad (1 point). **b** Designated detail from **a** in monochrome. The mucosa has been highlighted in red, the muscularis mucosae as an indicator for an intact, continuous submucosa in blue, the muscularis in yellow, and the serosa (only marginally present) in green. No layers have achieved continuity. Score points: 0 for first closed layer, 0 for count of healed layers, 0 since the epithelial layer has not closed. **c** the crypt architecture has not been restored (0 points). There is also no evidence of blood vessel ingrowth (0 points). **d** There is only occasional presence of fibroblasts or collagen formation (black arrowheads; both yield 1 score point). In contrast, there are confluent inflammatory cells (white arrows, 0 points). Total: 3 points. **e** Example of a well healed anastomosis. Overall healing has been rated good (3 points) and crypt architecture has been restored (1 point). **f** shows **b** in monochrome. The mucosa (red) and the serosa (green) have healed (4 point for first healed layer, 2 for total number of healed layers, 1 for closed epithelial layer). The muscularis mucosae (blue) and muscularis (yellow) are not continuous; however, this might be underscored here due to the large artificial tear (marked purple). **g** There is light scattering of blood vessels (white arrows, 2 points); fibroblasts and collagen fibers are confluent (black arrowheads, both 4 points). **h** Only occasional evidence of inflammatory cells (cluster marked with black arrowheads, 3 points) can be observed. Total points: 24. Magnification: **a, e, f**: 5 × ; **b, c**: 10 × ; **g, h**: 20 × ; **d**: 40 ×

Histological scoring of the anastomosis is one of the most objective and exact measures to evaluate the healing process. Here, performance of the microscopic sections and correct alignment of the anastomosis are pivotal. For the scoring of the healing process, Table 3 gives details about allocation of score points, and Fig. 4 demonstrates the application of this score. We recommend using this score in every experiment concerned with anastomotic healing, since it quantifies microstructural and cellular progress of healing and thus reflects the most accurate status of healing at the point of evaluation.

**Table 3:** Histological healing score

**Criteria**	**Score points (maximum = 29)**
Blood vessel ingrowth	0 = no evidence 1 = occasional evidence 2 = light scattering 3 = abundant evidence 4 = confluent cells or fibers
Fibroblasts	0 = no evidence 1 = occasional evidence 2 = light scattering 3 = abundant evidence 4 = confluent cells or fibers
Collagen formation	0 = no evidence 1 = occasional evidence 2 = light scattering 3 = abundant evidence 4 = confluent cells or fibers
Inflammatory cells	0 = confluent cells or fibers 1 = abundant evidence 2 = light scattering 3 = occasional evidence 4 = no evidence
First layer in which continuity has been restored (counted from the mucosa outwards towards serosa)	0 = no layer restored 1 = serosa 2 = muscularis 3 = submucosa 4 = mucosa
Number of healed layers	0 – 4 (none, mucosa, submucosa, serosa)
Epithelium closed	0 = no 1 = yes
Crypt architecture restored	0 = no 1 = yes
Overall healing quality	1 = bad 2 = normal 3 = good

#### Macroscopic assessment of the anastomosis

To classify the macroscopic aspects of anastomotic healing, combine the adhesion score with a classification of abscess formation.

**Fig. 5:**
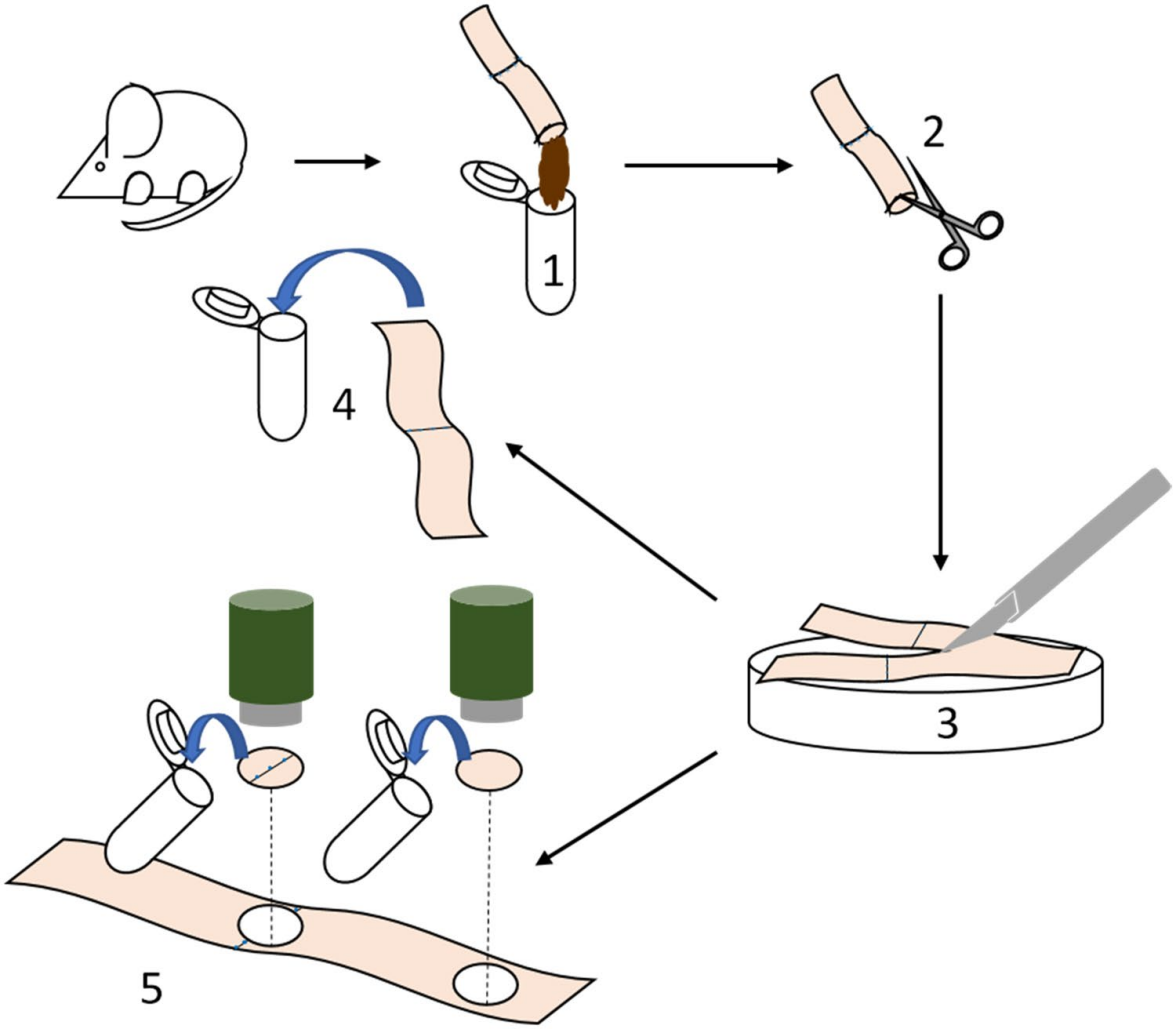
Preparation of samples. **1** After sacrificing the mouse, extract the colon, remove feces, and sample for microbiological analysis. Express the feces without disrupting the integrity of the colon using a swab. To increase the amount of material, the cecum can also be extracted and its content added to the feces sample. If a measurement of bursting pressure is required, take it before incising the colon. **2** Incise the colon lengthwise along the mesocolon, **3** flatten it onto a petri dish, and cut it into two halves using a scalpel. **4** Immerse one-half of the colon sample in tissue fixative for histopathological analysis. **5** From the other half, take two samples using a biopsy punch, one containing the anastomosis, the other far from the anastomosis

The adhesion score awards 0 to 7 points for adhesions; higher scores reflect a higher amount of adhesions (see Table 4 for details and Fig. 5a to d for a demonstration of how this score is applied).

**Table 4:** Adhesion score

**Criteria**	**Score points (maximum = 7)**
Are uterus, small intestine or omentum attached to the anastomosis?	1 point per adherent organ
Is any other organ attached to the anastomosis?	0 = no 1 = yes
Feasibility of removing the adhesions bluntly with a swab	0 = no adhesions in the first place 1 = all adhesions can be removed bluntly 2 = only part of the adhesions can be removed bluntly 3 = no adhesions can be removed bluntly at all

In addition, the abscess formation is scored in analogy to the clinical subclasses of stage II diverticulitis in the classification of diverticular disease – an established approach in clinical practice – by appending a letter a to c to the adhesion score (see Table 5 for details).

**Table 5:** Abscess classification

**Classification**	**Criteria**
A	No macroscopically visible abscess formation (note that this does not preclude micro-abscesses visible in histopathological examination of the tissue)
B	Limited, macroscopically visible abscess (“macro-abscess” for short)
C	Disseminated peritonitis and/or free anastomotic leakage and/or intestinal perforation

## Discussion

### Combining models for colitis and colorectal surgery

We demonstrate our combination model of experimental colitis and colorectal surgery as a feasible and adaptable setup for analysis of anastomotic healing during inflammatory conditions. Furthermore, we report standardized retrieval of biological samples and scores that describe the healing process.

Although historically most of the studies on anastomotic healing have been performed in rats and dogs [7], the intraabdominal intestinal immune response in mice is more comparable to that of humans [18]. Furthermore, mice are cheaper and easier to handle, and the wide variety of available knockout genotypes in mice offers the possibility to research the influence of an immense number of factors on anastomotic healing. Male mice lack the confounding influence of cycle-dependent sex hormones, whereas female mice allow for improved exchange of commensal bacteria through co-housing which is often not an option with the more aggressive males. The surgical approach has been designed to exclude ischemia as a confounding factor in development of anastomotic leakage by sparing all vessels, as research on anastomotic perfusion is not the aim of this model. In short, the model presented here enables researchers to isolate the effect of healing adverse processes like mucosal inflammation on intestinal surgery.

### Clinical examination

**Fig. 6:**
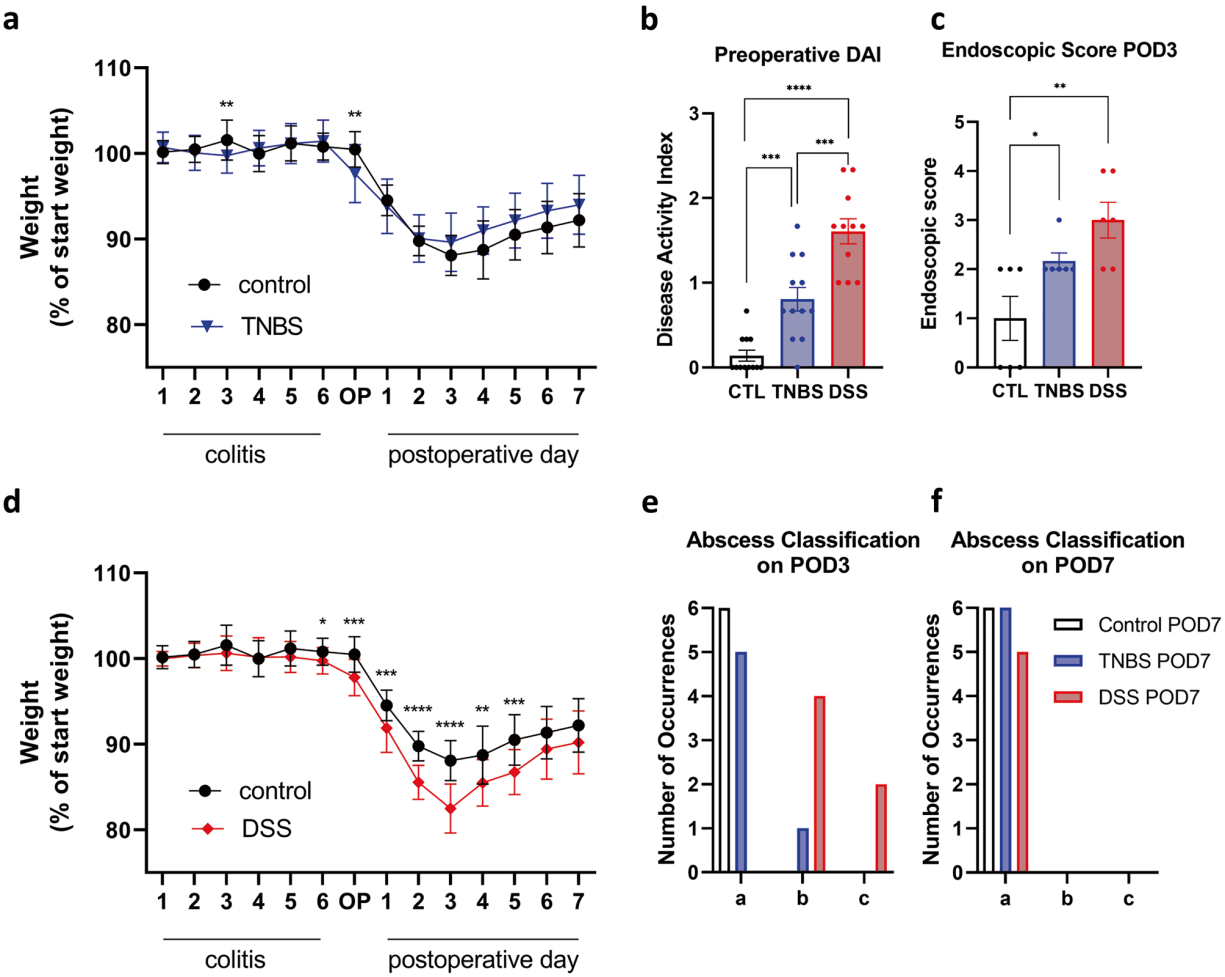
Exemplary evaluation data. **a, d** Weight curves showing body weight in % of start weight for each day after start of colitis, surgery day (OP), and postoperative days 1–7. **b** Preoperative disease activity index (DAI) for control group, TNBS group, and DSS group (n = 12). **c** Endoscopic score on POD3 for control group, TNBS group, and DSS group (n = 6). Results shown as mean ± SD for weight curves as mean ± SEM for bar graphs. Statistical differences determined by unpaired *t* test: *p ≤ 0.05, **p ≤ 0.01, ***p ≤ 0.001, ****p ≤ 0.0001. **e, f** Histograms of abscess classifications for control group, TNBS group, and DSS group (n = 6). Results shown in absolute number of occurrences per classification. One mouse in the DSS group had to be excluded from the POD7 evaluation due to meeting the abort criteria before evaluation. The difference between the control and DSS group on POD3 is significant on a 99.5% confidence level (exact Fisher test for three categories)

Weight loss can be used as a surrogate parameter for postoperative recovery. It is especially helpful in evaluating the systemic effect of colitis on the anastomotic healing process. The control groups (See Fig. 6a, d) which have undergone surgery without any colitis present show a characteristic, constant weight loss of up to 15% of their initial weight. They reach the nadir at POD3 after which they start to slowly recover. In combination with perioperative DSS colitis, we have observed weight loss to be more severe and start preoperatively, whereas TNBS colitis does not seem to affect the weight as DSS colitis (see Fig. 6a, d). However, it is only a rough health indicator for an individual. For example, excessive weight loss in an animal may be attributed to reduced food and water intake caused by postoperative pain, colitis, and intestinal paralysis due to analgesics or surgery. If, however, these factors are taken into account and weight loss is considered in combination with other markers of the healing process such as bursting pressure measurements, endoscopy, and macroscopic and microscopic assessment of the anastomosis, it provides an easily measurable parameter which can be very useful for quick orientation on the mouse’s health.

### Bursting pressure measurement

The rationale behind bursting pressure measurements is the aim to functionally characterize the stability of the anastomosis. The measurements can give an impression on the stability as well as the completeness of the healing process. The fluid instillation will reveal anastomoses prone to leakage or those that have insufficient healing. However, the measurements can easily yield false values: until the anastomosis has gained back enough tensile strength, success of the healing process is entirely dependent on the mechanical apposition by suture [14] or staples – this is the case during the first 3 to 5 days after surgery [19] due to the high collagenase activity resulting in a reduction of strength of up to 70% within the first 48h [8]. That means, this method will mostly reflect mechanical stability of the sutures instead of the progress of healing, and the suturing technique may mask severe healing defects. Additionally, the relevance of non-physiological bursting pressures of, e.g., more than 100 mmHg or bursting sites in places other than the anastomosis itself (especially in the early healing stages) remains questionable, as intraluminal pressure is usually low. They might be a better indicator for high mechanical stability of the sutures rather than reflect the state of the healing process. Furthermore, the lumen at the anastomotic site is often constricted, therefore reducing its radius. This might interfere with the comparability of bursting pressures recorded at the anastomosis and not at the anastomosis, thus further undermining the relevance of bursting pressure measurements. We therefore caution against using this method as a sole determinant of healing and recommend taking into account whether the bursting occurs directly at the anastomosis or in another region of the intestine. For bursting directly at the site of anastomosis, we recommend considering only pressures which are lower than physiological intraluminal pressure.

### Endoscopy scoring

Endoscopy is already widely established for diagnosis and treatment of anastomotic leakage in human patients. It is an easy method to measure macroscopic healing from the luminal side. If available, additional fluorescence endoscopy of labeled markers can be used to assess inflammation in the anastomotic tissue [17]. We recommend using this score in any experiment on anastomotic healing because of the high relevance of endoscopy in clinical practice. The scoring system presented here reduces the complexity of potential findings during endoscopy to a standardized, simple score which could be shown to be a valid parameter for the healing process (see below).

### Histological score

In order to get a measure of the microstructural and cellular changes, we have modified the established scoring system proposed by Phillips et al. [20]. The original score focuses on the presence of inflammatory cells and fibroblasts, progress of collagen formation, and ingrowth of blood vessels. On a molecular level, collagen deposition has been viewed as one of the most reliable criteria for measurement of the process of anastomotic healing [21]. We have added some criteria which consider the tremendous relevance of structural integrity and mechanical stability for healing. Namely factoring in a count of the layers that have completely healed, the first layer in which continuity has been restored, whether the epithelium has completely sealed the defect, and whether crypt architecture has been restored. This accounts for the importance of healing on a microscopic and cellular level and objectivizes these findings. We recommend using this adapted score, because it can serve as an excellent primary outcome to evaluate quality of healing.

### Macroscopic assessment of the anastomosis

We have developed a combined adhesion and abscess score for standardized reporting of macroscopic aspects of anastomotic healing. This dual scoring system allows to record descriptive data on the conditions within the peritoneal cavity upon evaluation.

However, the limitations of this score must be considered. The mechanisms of adhesion formation and the role of adhesions in intestinal healing are still huge fields of research with many unknowns. Lacking a proven causal relation between adhesion formation and anastomotic healing, the combined adhesion score is not suitable as a surrogate parameter for healing or as a parameter to validate other findings against.

However, it can be used as a means of taking standardized notes of the conditions found upon dissection of the euthanized mouse. Keeping data in this form could be very useful as the basis of research projects which examine the role of adhesions and abscess formation in the context of anastomotic healing.

### Expected results

To validate our model, we here present data from an experiment comparing severe DSS colitis, mild TNBS colitis, and a control group without colitis as preoperatively evaluated by DAI (Fig. 6b). The endoscopic healing score on POD3 correlated with preoperative colitis severity (Fig. 6c). There is no significant difference in the weight curves postoperatively comparing the control and TNBS group; however, with severe DSS colitis, postoperative weight loss was significantly higher compared to the control group (Fig. 6a, d). For exemplary data using the histological score, we refer to our previously published study reporting on the use of Ac2-26 coated nanoparticles improving anastomotic healing in a DSS colitis model [22]. In our experiments, both the DSS and TNBS model did not produce significant differences in adhesion formation between the experimental and control groups on any POD.

The abscess classification on the other hand did show a significant difference between the different models of experimental colitis (see Fig. 6e, f). The TNBS and control groups did not show any abscess formation at POD3 or POD7. The DSS group, however, exhibited a significantly higher occurrence of abscess formation on POD3, whereas on POD7, there was no more difference between the groups.

In summary, this data suggests that DSS colitis impairs especially the early healing phase through a more systemic inflammation, whereas TNBS colitis disrupts anastomotic healing more locally and produces less leakage. Depending on the desired effect, either of these models can be chosen.

## Conclusion

In summary, DSS and TNBS colitis are widely used models of experimental colitis and can be well combined with surgery, as they lead to timed and reproducible severity of inflammation. Different techniques of anastomoses can be used, depending on the intended scope of research (leakage or sufficient healing model). Different scores aim to improve the quality of reporting by standardizing results and thus reduction and refinement of animal experiments. The bursting pressure as an established functional outcome has to be questioned, whereas an endoscopic score to assess healing from the luminal side of the colon in vivo via murine colonoscopy valuable measures of luminal healing. A refined version of the histological score to assess microscopic healing can be used to examine cellular components of healing and microstructural progress in detail. Tissue can be further processed using molecular analysis.

Ultimately, this model offers the possibility to optimize evaluation of strategies to improve anastomotic healing and treatment of its complications.

## Supplementary information

Below is the link to the electronic supplementary material.

Supplementary file1 (DOCX 14468 KB)
