# Supplementary material for: Intestinal anastomotic healing models during experimental colitis
Source: Int J Colorectal Dis. 2021 Aug 28;36(10):2247–59. doi: 10.1007/s00384-021-04014-5 (PMC8426221; doi:10.1007/s00384-021-04014-5)
Supplement: Supplementary file 1 — Supplementary file1 (DOCX 14468 KB) [file 384_2021_4014_MOESM1_ESM.docx]

# Supplemental Material

Intestinal anastomotic healing models during experimental colitis.

**Authors**

**J. R. E. Miltschitzky^1,^*, Z. Clees^1,^*, M.-C. Weber^1^, V. Vieregge^1^, R. L. Walter^1^, H. Friess^1^, S. Reischl^2,+^, P.-A. Neumann^1,+^**

**Affiliations:**

^1^ Technical University of Munich, School of Medicine, Klinikum rechts der Isar, Department of Surgery, Munich, Germany, ^2^ Technical University of Munich, School of Medicine, Klinikum rechts der Isar, Department of Diagnostic and Interventional Radiology, Munich, Germany

*^,+^ These authors contributed equally.

**Corresponding author:**

Dr. Philipp-Alexander Neumann, MD

Department of Surgery

Klinikum rechts der Isar

Technical University of Munich

Ismaningerstraße 22

D-81675 Munich

Germany

Email: Philipp-Alexander.Neumann@tum.de

Content

[1. Materials 2](#_Toc67314525)

[2. Step-by-step protocol recommendations 3](#_Toc67314526)

[2.2 Monitoring and daily chores 3](#_Toc67314527)

[2.3 Special tasks during induction of colitis 4](#_Toc67314528)

[2.4 Anaesthesia 4](#_Toc67314529)

[2.5 Analgesia 5](#_Toc67314530)

[2.6 Endoscopic assessment of the mucosa and/or site of anastomosis 5](#_Toc67314531)

[2.7 Colorectal anastomosis surgery 6](#_Toc67314532)

[2.8 Bursting pressure measurement 11](#_Toc67314533)

## Materials


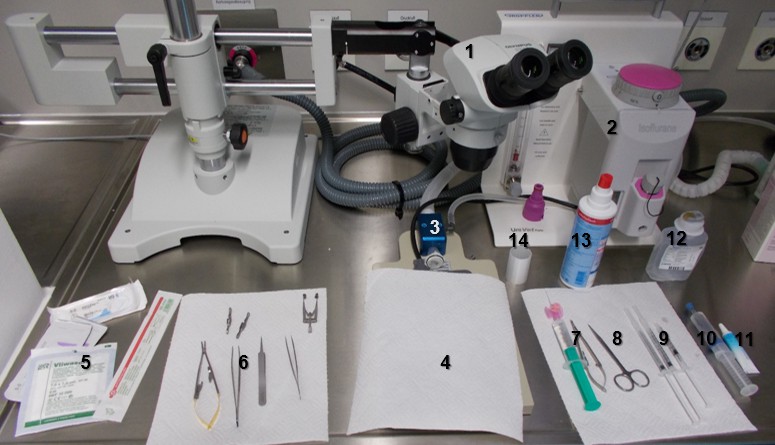


**Fig. S1: Overview of the operation set-up.** 1) Operating microscope, 2) isoflurane vaporizer 3) respirator mask for mice 4) heating pad 5) consumables: gauze, swabs, sutures 6) surgical instruments 7) irrigation 8) surgical instruments 9) pain medication 10) gel 11) eye ointment 12) 0.9% sodium chloride solution 13) disinfectant 14) adhesive tape.


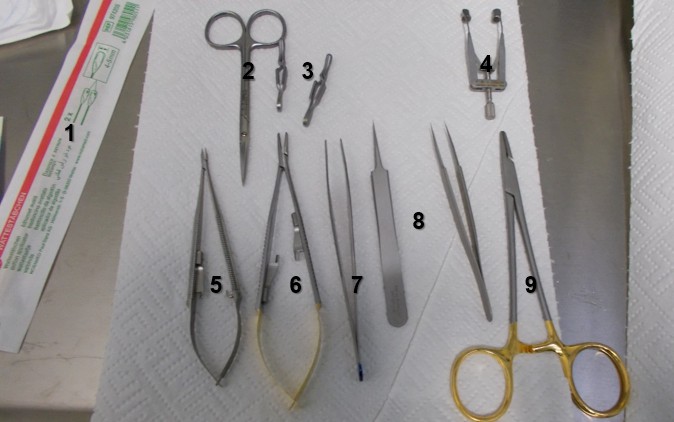


**Fig. S2: Surgical instruments.** 1) Cotton swabs 2) scissors 3) bulldog serrefine clamps 4) retractor 5) small needle holder 6) medium sized needle holder 7) coarse pincers 8) two fine pincers 9) coarse needle holder. All surgical instruments were bought from Fine Science Tools, Heidelberg, Germany. For anastomosis, 9-0 sutures and for skin, 5-0 sutures are used.


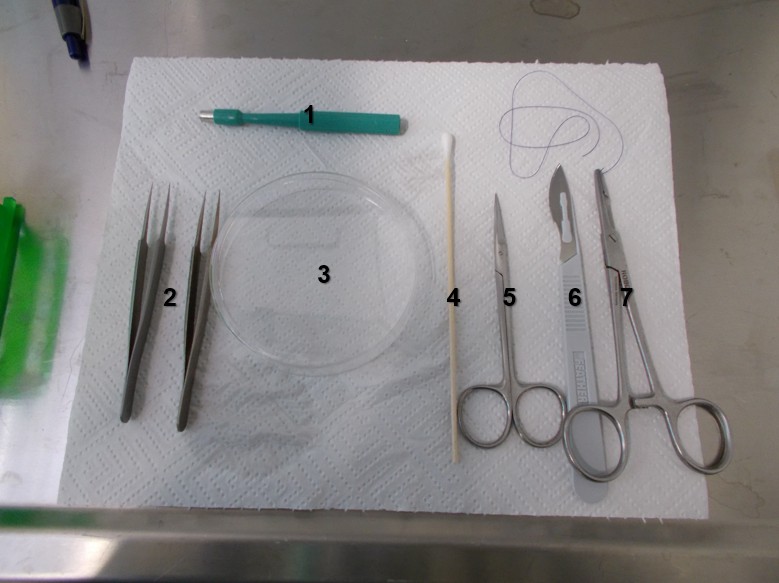


**Fig. S3: Instruments for evaluation.** 1) Biopsy punch 2) two fine pincers 3) petri dish 4) swab 5) scissors 6) 22-blade scalpel 7) coarse needle holder with 4-0 ligature.

## Step-by-step protocol recommendations

### 2.2 Monitoring and daily chores

In addition to analgesia administration, score mice daily based on clinical aspects of post-operative healing as well as national and lab-internal animal welfare regulations. We recommend a scoring protocol including the criteria listed in Table S1. Define an algorithm for therapeutic measures and clear endpoint criteria (i.e. substitution of s.c. fluid and analgesia at a total score of 3, endpoint reached at total of 8 score points or any single score of 4).

**Table S1: Scoring protocol for daily assessment**

| **Criteria** | **Score points** |
| --- | --- |
| Weight | 0 = no reduction  1 = reduction of 0 – 5%  2 = reduction of 6 - 10%  3 = reduction of 11 – 19%  4 = reduction of >19% |
| Fur | 0 = normal, shiny, smooth  1 = piloerection |
| Behavior | 0 = normal  1 = subdued, no exploration, reduced interaction  4 = apathy, isolation, stereotypical behavior |
| Posture | 0 = normal  2 = intermittent cowering or shivering  4 = permanent cowering or shivering |
| Pain | 0 = no indication  2 = defensive behaviour upon palpation |
| Impaired wound healing | 0 = no indication  2 = irritated or oozing wound  4 = dehiscent suture or open abdomen |
| Dehydration | 0 = skinfolds straighten out within 2s  1 = persistent skinfolds |
| Mucous membranes (ear, skin, extremities) | 0 = rosy  1 = pale |
| Stool | 0 = formed  1 = diarrhoea |

### 2.3 Special tasks during induction of colitis

During induction of DSS colitis, the continuous uptake with drinking water provokes a mid-grade inflammation of the intestinal mucosa, which will be monitored by daily determination of the disease activity index (DAI). A first reaction of the intestinal mucosa can be expected after two days. Weigh the animals daily, examine the stool regarding its consistency and test it for occult rectal bleeding (hemoccult-test). Target a DAI lower than 1.

### 2.4 Anesthesia

Since isoflurane anaesthesia cannot provide a sufficient analgesic effect for surgical tolerance, combine it with oral and/or intraperitoneal analgesia.

Immerse the mouse completely into a chamber that is then flooded with 2 - 5% of isoflurane with 3L/min of oxygen as a carrier gas. Observe transition through Guedel’s excitation state carefully and test positional reflexes by gently tipping the chamber to roll the mouse on its backside. If no reflectory rolling back into a prone position can be seen, take the mouse out of the chamber and place it onto a pad for the planned procedure. Insert its nose into the respirator mask and maintain anaesthesia with 1.5 - 2% of isoflurane at an oxygen flow of 1L/min. To protect the cornea, eyes are covered with eye ointment (see Fig. 4). Surgical tolerance must be tested by checking the paw reflex: absence of reflectory withdrawal of the extremity if the mouse’s hind paw is squeezed with blunt pincers indicates sufficient anaesthesia. Take care not to injure the paw.

Note: The animal’s natural temperature regulation is suppressed under general anaesthesia. To avoid hypothermia, use a heating pad. However, the tail is particularly sensitive to heat, which can cause postoperative oedema and necrosis. To avoid these complications, isolate the tail by cellophane or cork to reduce local temperature to between 26 to 30°C or use an adjustable heating pad.

Post-operatively, isoflurane anaesthesia does not need to be antagonized. For better postoperative recovery, gently hold the mouse in your hands to warm it while awakening from anaesthesia.


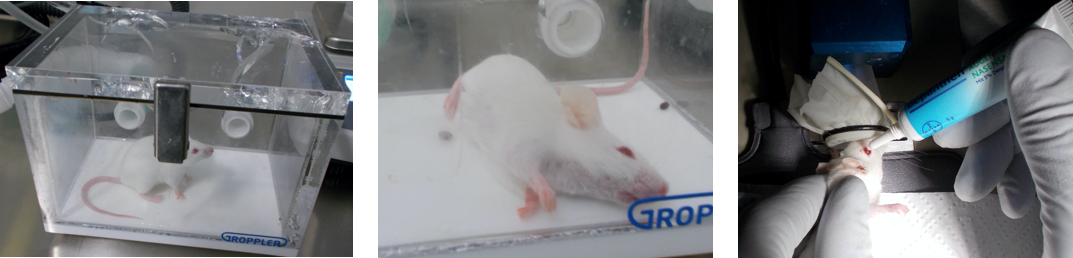


**Fig. S4: Induction of anesthesia and application of eye ointment.**

### 2.5 Analgesia

For surgery, administer 1 mg/kg of Metacam s.c. 20 minutes prior to isoflurane application, and up to three injections of buprenorphine 0.1 mg/kg s.c. on the day of surgery. Administer an additional dose of 1 mg/kg live weight of Metacam s.c. each on the first and second post-operative day.

### 2.6 Endoscopic assessment of the mucosa and/or site of anastomosis

Assess mucosa and/or anastomosis directly *in vivo* before surgery and/or before evaluation. After anaesthesia, place the mouse onto the heated operating pad in a prone position. Lubricate the anus with a drop of local anaesthetic gel (e.g. Instillagel®). Insert the endoscope under careful insufflation of 0.9% saline solution (see Figure 5). For standardized video documentation, the endoscope is advanced up to the colic flexure, then the video is started to record the entire colon starting at the flexure while pulling back steadily until exiting from the anus. Alternatively, air-assisted endoscopy can be performed by using a small pump, connecting it to the endoscope and regulating air-flow manually.


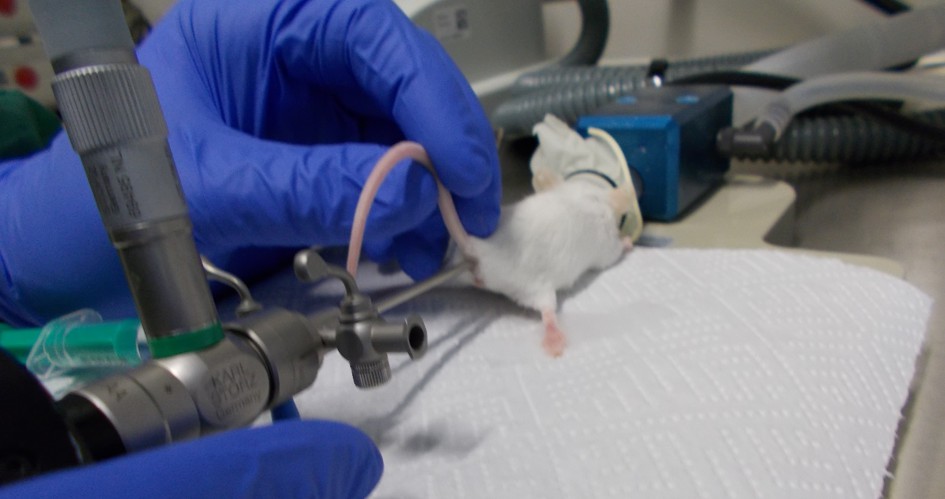


**Fig. S5: Colonoscopy.** Mouse in prone position, nose in the respirator. The stiff murine colonoscope is inserted and advanced up to the colic flexure.

### 2.7 Colorectal anastomosis surgery

Note of caution: All contact with any viscera must be performed bluntly, using only swabs and anatomic forceps to avoid damaging the fragile tissue. No part of any organ may be squeezed or incarcerated at any time during the procedure. To avoid viscera from drying out and to prevent intraoperative loss of bodily fluid, provide intermittent irrigation by applying 0.9% sodium chloride solution into the open abdominal cavity during the complete procedure. In addition, apply about 1 ml of 0.9% sodium chlorine solution intraperitoneally before closing the peritoneum.

Place the mouse in supine onto the heating pad and fixate the extremities with adhesive tape. Use an operating microscope with 10-fold magnification. Shave and disinfect the mouse’s abdomen before skin incision. To open the abdominal cavity, start with a medial laparotomy of 1.5 to 2 centimetres by dissecting first skin and attached subcutaneous fat with scissors. Next, open the peritoneum along the linea alba to preserve abdominal muscle using the same scissors (see Fig. 6).

This approach allows for minimal bleeding during preparation. Insert a retractor for good visibility. After laparotomy, mobilize the small intestine and cecum out of the abdominal cavity and wrap them into moistened gauze dressings. The recto-sigmoidal part of the large intestine can be identified by using the inferior pole of the left kidney as a landmark.

The mesocolon should face to the right. Mobilize the mesenterial artery and pierce the vessel-free part of the mesocolon in between two branches using fine pincers to create a hole (see Fig. 7). Insert scissors into this gap and transect the colon. Do not resect any part of the colon, mesocolon or the vessels to avoid confounding the experiment with potential ischemic components of anastomotic leakage (see Fig. 8).

In case of bleeding, haemostasis can be achieved in most cases by compression with swabs. Reconnect the two separated ends of the intestine in an end-to-end anastomosis. Depending on the requirements of the experiment, the colonic stumps can be connected using running sutures or single stitches of varying distances. More stitches and running sutures provide tighter approximation of the anastomotic region. Calibrate the mechanical stability of the anastomosis to the desired level. To get a high rate of AL, number of stitches can be reduced to 8, while 12 single sutures usually guarantee a very low leakage rate. We recommend using monofilamentous, absorbable polyglactin or polydioxanone 9-0 sutures. First, anchor the anastomosis with two stitches, one on the mesenterial side of the anastomosis, the other on the opposite side. Leave one end of each of these sutures long after knotting and insert it into a bulldog serrefine clamp (see Fig. 8). Complete the ventral row with running or single stitches as needed for your experiment. Now disengage the anchor sutures from the clamps and flip the colon around the axis of the meso to expose the unfinished half of the anastomosis. This is best done by pushing the end of one anchor suture through the hole in the meso between mesenteric vessels and colon and grabbing it from the other side. Now insert the anchor sutures into the serrefine clamps again and complete the dorsal row like the ventral one previously. Trim the sutures and reposition all viscera anatomically into the abdominal cavity (see Fig. 9). Control the situs for any bleeding and apply 1 ml of 0.9 % sodium chlorine solution before closing first peritoneum, then skin (see Fig. 10). We recommend suturing both peritoneum and skin with running 5-0 non-resorbable, monofilamentous sutures. The approximate duration for surgery is about 30 – 45 minutes.


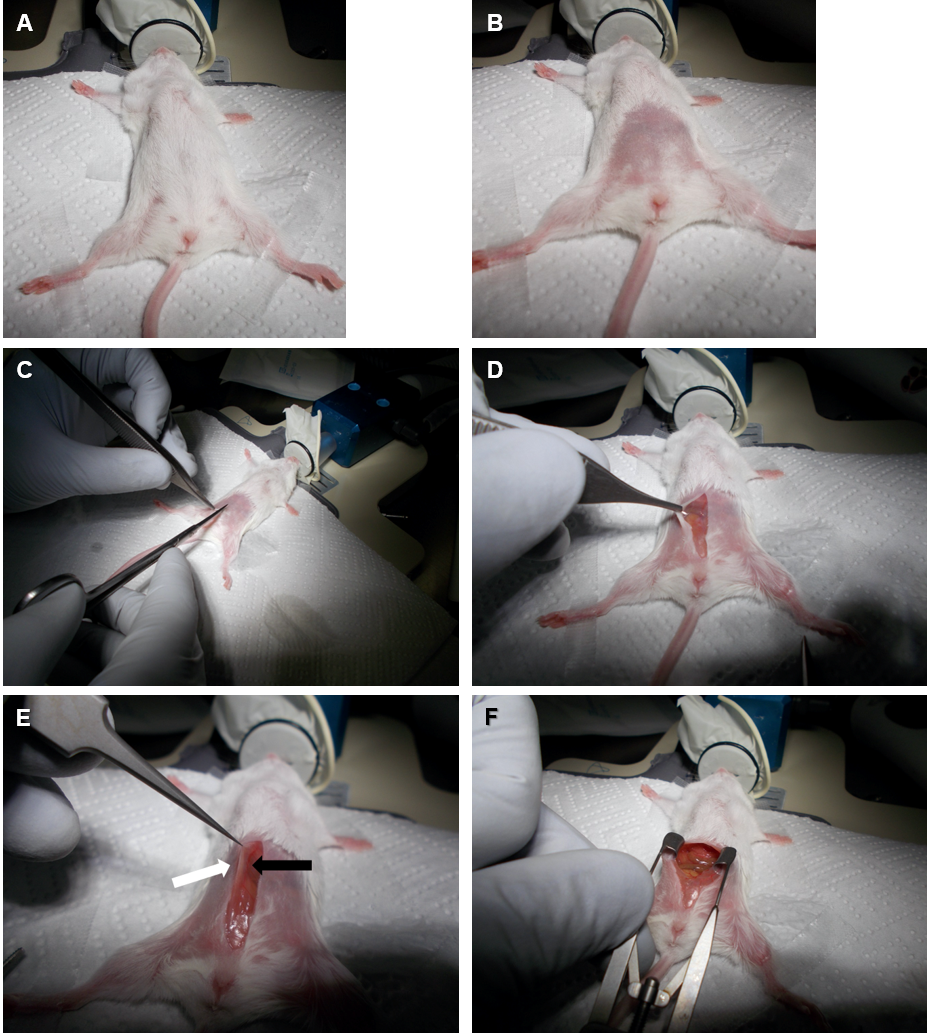


**Fig. S6: Surgical procedure – Part 1.** A) Mouse fixated onto heated pad in supine spread-eagled position. The pad temperature has been regulated by layers of cellophane to avoid tail complications. B) shaved abdomen. C+D) skin incision. E) The linea alba (white arrow, lifted up in pincer) needs to be identified and incised to avoid injury to the epigastric vessels (left epigastric vessels marked by black arrow). F) After peritoneum incision insertion of the retractor provides necessary visibility.


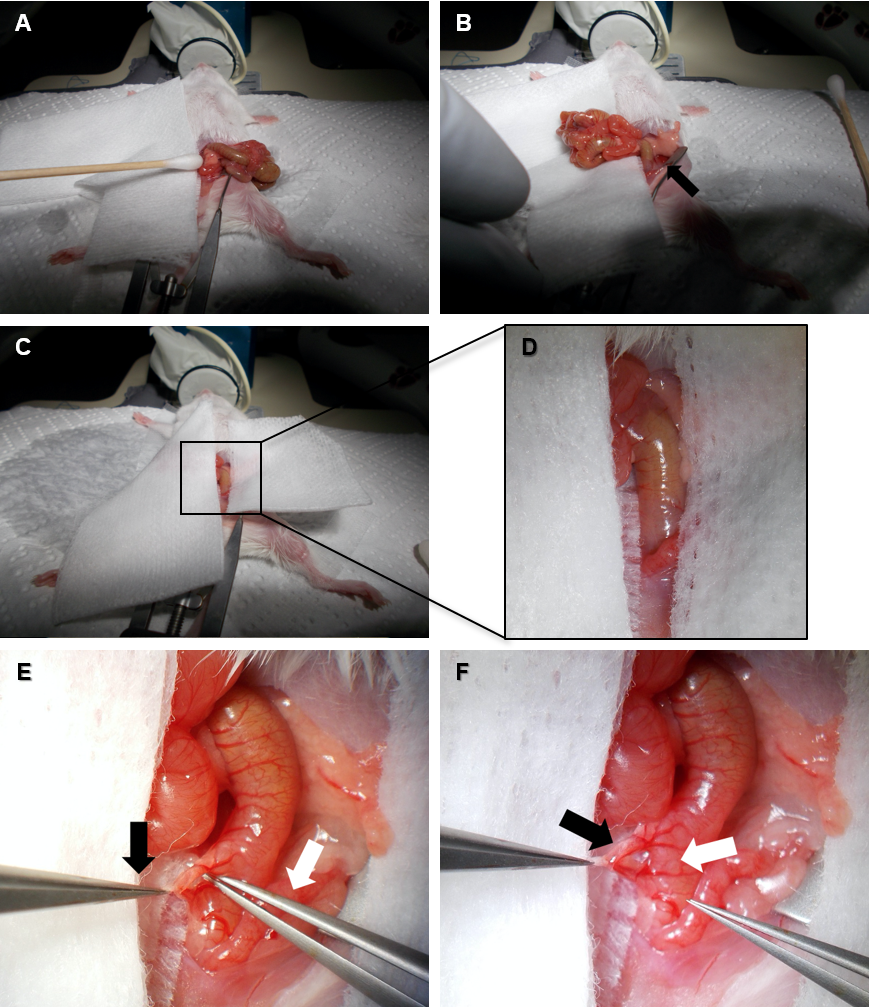


**Fig. S7: Surgical procedure – Part 2.** A) Small intestine and cecum have been mobilized to the left. B) Viscera are then brought to the right side and placed onto a moist gauze dressing for support and protection. Note the position of the lower left kidney pole (black arrow), which serves as orientation for the incision of the colon at the recto-sigmoidal transition later. C) Place a second sheet of moistened gauze on the left side of the incision, cover all viscera leaving only the relevant part of the colon exposed. D) View through operating microscope. E) A hole is opened in the membranous part of the mesocolon between the vessel arcades while pincers lift the mesocolon (black arrow) and another pair of pincers is burrowing (white arrow). F) This leaves a gap between the mesenterial artery (black arrow) and the colon (white arrow), into which the scissors can be inserted for transection. CAUTION: the pincers can injure vessels and cause severe bleeding! Only touch and manipulate the fatty and membranous parts of the mesocolon.


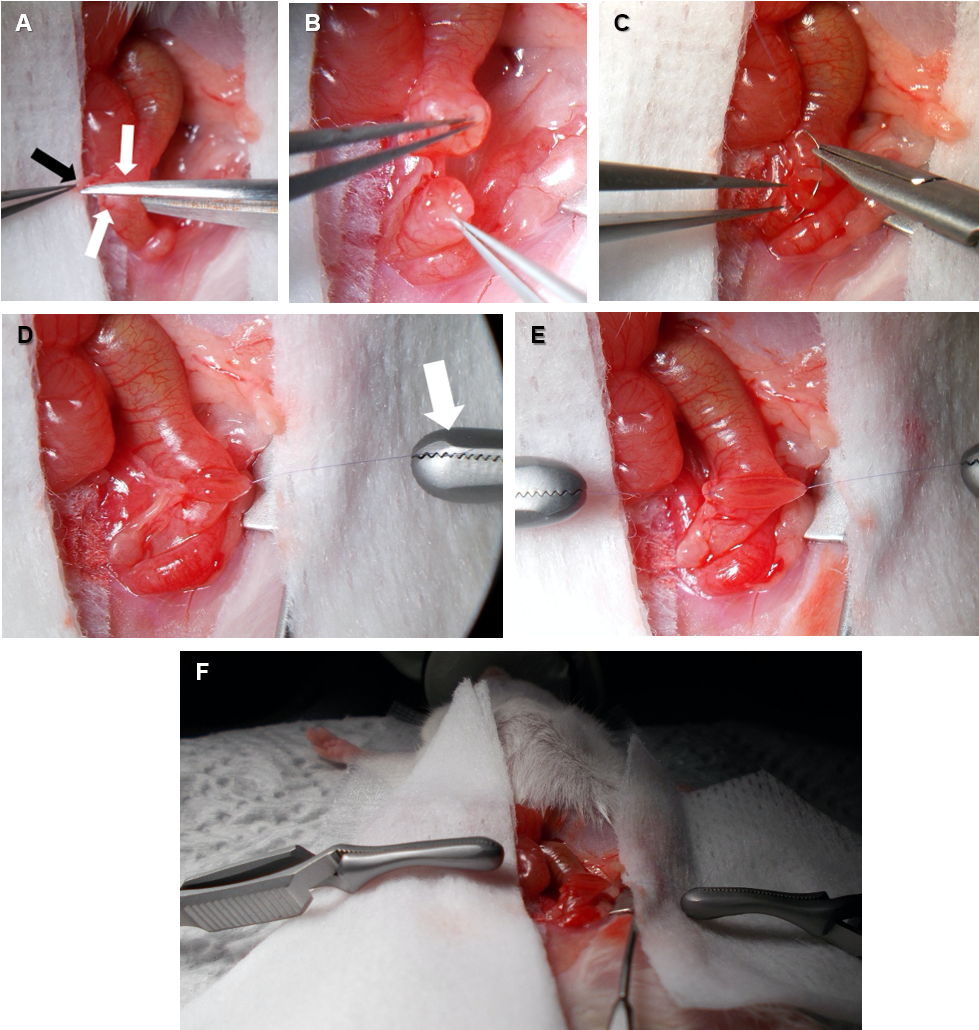


**Fig. S8: Surgical procedure – Part 3.** A) One blade of the scissors is carefully pushed under the colon and through the gap in the mesocolon. Ensure thatthe mesenterial artery (black arrow) is not caught between the blades and that the incision is parallel to the arcade vessels (white arrows) to avoid any injury to the vessels resulting in bleeding or ischemia of the tissue. B) After transection, contraction of the circular muscles of the colon stumps is normal. Lack of bleeding from the stumps as seen in this photo is the benchmark sign of a clean incision without injury to the vessels. C) First anchoring suture is placed using a fine needle holder and a 9-0 Vicryl suture. D) After tying, one suture end is left long and attached to a bulldog serrefine (white arrow). E) In the same fashion, the second anchoring suture is tied and attached to a bulldog serrefine clamp. F) Direct view of operating site without microscope.


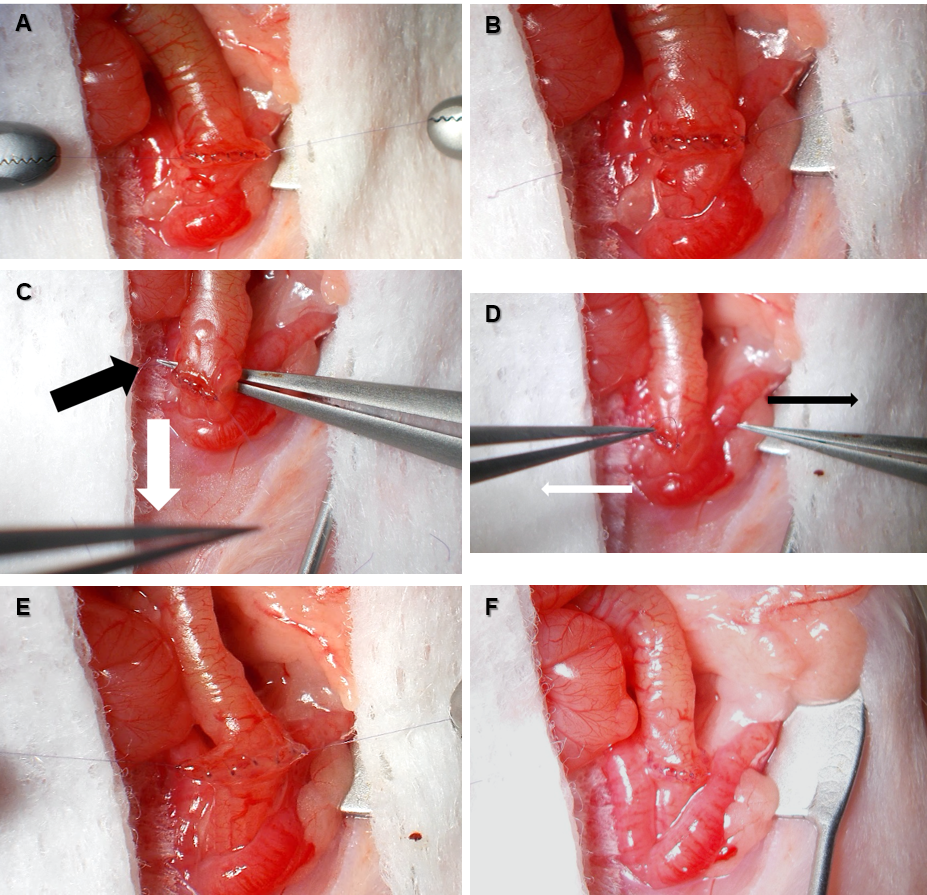


**Fig. S9: Surgical procedure – Part 4.** A) By placing 5 stitches equally spaced in between the two anchors, the ventral row is completed. The tendency of the colonic tissue to form everted "lips" at the site of the anastomosis needs to be counteracted by guiding the needle on a course tangential to the wall of the colon when suturing. B) For the "flipping manoeuvre” detach the bulldog serrefine clamps. C) Then the colon is lifted up by grabbing the anti-mesenterial anchor with fine pincers held in the left hand (white arrow) and inserting the right-hand-set of pincers through the gap in the mesocolon to grab the mesenterial anchor (black arrow). D) After getting a hold of the mesenterial anchor, the colon can be flipped over by pulling the mesenterial anchor to the left, through the gap in the mesocolon (direction of the slim black arrow) while the anti-mesenterial anchor is pulled to the right (direction of the slim white arrow), thus exposing the dorsal side of the anastomosis. E) The dorsal row is completed with 5 stitches exactly like the ventral one, adding up to a total of 12 stitches. If the perfect spacing cannot be achieved, the number of stitches can be adapted to a number between 10 and 14 stitches as needed to provide regular spacing. F) Finished anastomosis after shortening the two anchor sutures.


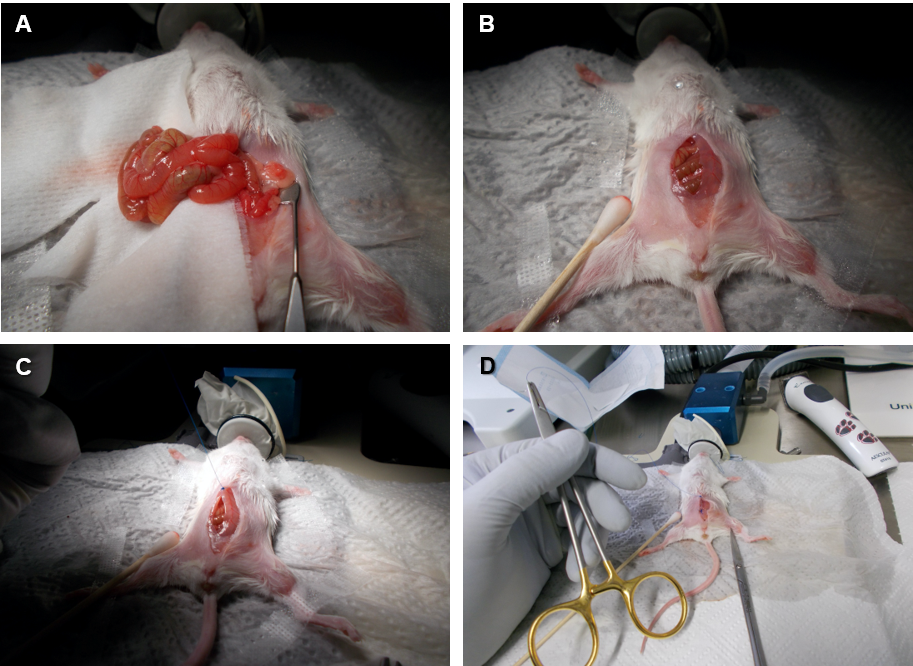


**Fig. S10: Surgical procedure – Part 5.** A, B) Unwrapping and blunt repositioning of the luxated viscera. C) The peritoneum is closed with a running suture using 5-0 Vicryl. D) The skin is closed in the same fashion.

### 2.8 Bursting pressure measurement


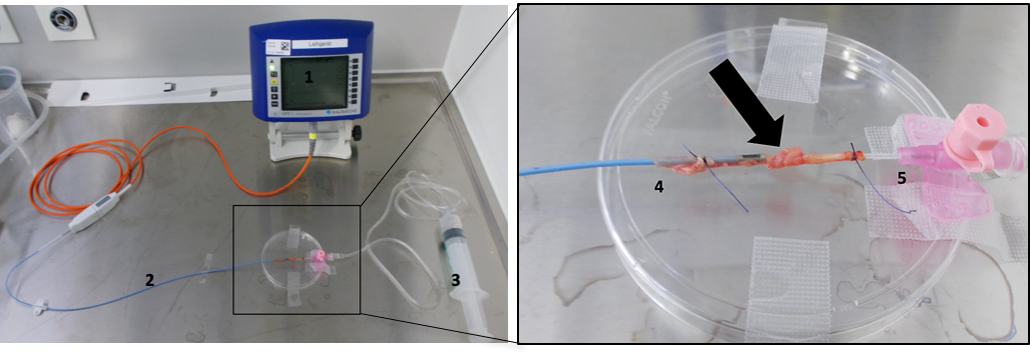


**Fig. S11: Bursting pressure measurement.** Mount the colon onto a petri dish with a plastic cannula inserted into the lumen on the aboral side of the anastomosis and an ICP probe on the oral side. Both sides are fixated to the probes with 4-0 ligatures. After calibrating the pressure in the colon to 0 mmHg, fill it with isotonic saline solution using a syringe attached to the cannula until rapid pressure drop while constantly measuring the intraluminal pressure. Record the maximum of the pressure spike as bursting pressure. 1) pressure monitor, 2) ICP probe, 3) syringe, infusion tube and plastic cannula, 4) ICP probe inserted into the oral side of the colon, 5) cannula inserted into the aboral side of the colon (black arrow).
